# Data-driven precision: artificial intelligence redefining immunoradiotherapy in advanced pancreatic cancer

**DOI:** 10.3389/fphar.2026.1804673

**Published:** 2026-05-08

**Authors:** Yao-Wen Liu, Xiao-Ding Men, Bin-Ru Di, Yu-Lin Lei, Li Xiang, Yu-Hao Luo

**Affiliations:** Department of Oncology, The Affiliated Hospital of Southwest Medical University, Luzhou, China

**Keywords:** artificial intelligence, immunoradiotherapy, multimodal data integration, pancreatic ductal adenocarcinoma (PDAC), tumor microenvironment

## Abstract

Advanced pancreatic ductal adenocarcinoma (PDAC) remains among the most formidable challenges in oncology, driven by a profoundly immunosuppressive tumor microenvironment (TME) and pervasive resistance to systemic and local therapies. Although immune checkpoint inhibitors (ICIs) can synergize with radiotherapy (RT) in several malignancies, the clinical benefit of immunoradiotherapy (iRT) in PDAC has been modest, highlighting the limitations of population-averaged paradigms that fail to capture extensive inter- and intratumoral heterogeneity. Here, we synthesize an artificial intelligence (AI)–enabled framework to refine both the biological rationale and clinical implementation of iRT for advanced PDAC through integrative analysis of multimodal data (clinical variables, imaging, RT dose distributions, and multi-omics). We highlight advances in three domains. First, AI-based deconvolution of TME heterogeneity can delineate clinically relevant molecular subtypes and spatial immune architectures that may be therapeutically tractable. Second, AI-driven modeling can optimize spatiotemporal RT–immunotherapy interactions, informing individualized dose, fractionation, and biologically guided target definition. Third, AI-supported predictive modeling and adaptive feedback can enable response-guided treatment adjustment beyond static planning. We also discuss unresolved clinical questions and key translational barriers, including data scarcity, lack of standardization, and limited interpretability. Finally, we outline priorities for translation—prospective digital biobanks, hybrid mechanistic–data-driven modeling, and adaptive trial designs—to enable rigorous validation and clinical deployment. Collectively, these developments position AI as a catalyst to move iRT for PDAC from empiricism toward real-time, individualized precision medicine.

## 1 Introduction

Pancreatic ductal adenocarcinoma (PDAC) has an exceptionally poor prognosis and is now the third leading cause of cancer-related mortality in Western countries, with projections that it may rise to second within the next decade (Aronsson et al., 2019; Chandana et al., 2024). Because most patients present with locally advanced or metastatic disease, systemic chemotherapy remains the clinical backbone; however, benefit is modest and often offset by substantial toxicity (Mastrantoni et al., 2024; Meneses-Medina et al., 2022; Chin et al., 2018). In contrast to melanoma and lung cancer, where immune checkpoint inhibitors (ICIs) targeting the PD-1/PD-L1 axis have reshaped outcomes, PDAC typically exhibits limited responsiveness and is widely viewed as a prototypical “immune-desert” tumor (Burrack et al., 2019; Jin et al., 2024; Rao et al., 2020; Elias et al., 2018).

Radiotherapy (RT), as an established local modality, has therefore been explored as a strategy to augment immunotherapy (Kroeze et al., 2023). Mechanistically, RT can induce immunogenic cell death, increase antigen release and presentation, enhance vascular permeability, and remodel the local cytokine milieu—changes that may “heat up” immunologically cold tumors and broaden systemic antitumor immunity (Sundahl et al., 2025; Zhou et al., 2021; Geng et al., 2021). Yet clinical outcomes have been inconsistent. Trials of immunoradiotherapy (iRT) in advanced PDAC have generally delivered response rates and survival benefits below expectations, underscoring substantial between-patient heterogeneity (Ma et al., 2025; Tyler et al., 2020).

This discordance reflects the biological complexity of PDAC and the limits of population-averaged treatment–response models. PDAC is shaped by a distinctive genomic landscape, dense desmoplastic stroma, complex immune networks, and profound metabolic reprogramming, all of which evolve over time and under therapeutic pressure (Hb et al., 2021). Overcoming these barriers requires a methodological framework that can represent the TME as a multidimensional, spatially organized, and dynamic system, enabling more reliable response prediction and rational personalization.

**FIGURE 1:**
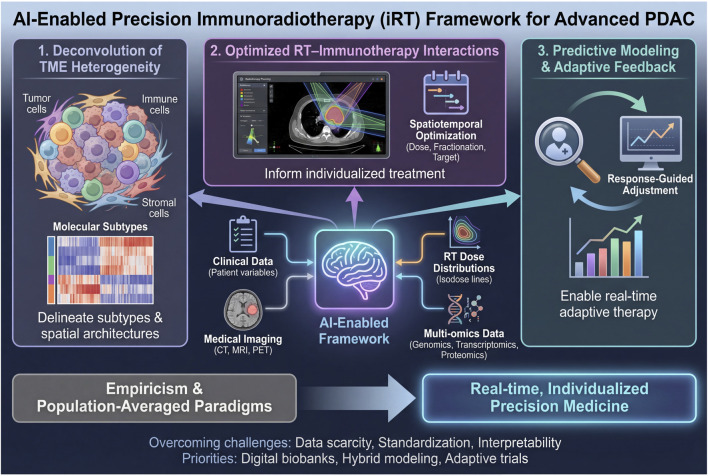
AI-assisted integration of clinical data, imaging, RT dose maps, and multi-omics to support planning and longitudinal assessment of immunoradiotherapy in advanced PDAC.

Artificial intelligence (AI), particularly machine learning and deep learning, is well suited to this task because it can integrate high-dimensional, nonlinear signals across heterogeneous data types (Jabbari and Rezaei, 2019; You et al., 2022). Available evidence in this area is heterogeneous and remains largely retrospective, with limited PDAC-specific prospective validation. Against this background, we summarize how AI-assisted integration of multi-omics, imaging, digital pathology, and clinical data may support risk stratification, RT planning, and longitudinal response assessment in advanced PDAC (Figure 1).

## 2 AI-driven deep decoding of the tumor microenvironment: beyond traditional classification

Heterogeneous iRT response in PDAC is largely rooted in extraordinary TME diversity. Conventional pathology and bulk molecular subtypes (e.g., classical–basal) capture only coarse averages and often miss spatial and temporal structure. By integrating multimodal data, AI enables a more granular, spatially resolved, and functionally meaningful dissection of the PDAC ecosystem.

### 2.1 Multi-omics integration and discovery of novel subtypes

AI-based unsupervised learning (deep clustering, autoencoders) can jointly analyze genomic alterations (mutations, copy-number variation) alongside transcriptomic, epigenomic, proteomic, and metabolomic profiles to uncover latent biological programs that are difficult to detect with conventional approaches (Duo et al., 2024; Chan et al., 2023; Sharma et al., 2024). Graph neural networks (GNNs) further encode genes or proteins as nodes and interactions as edges, allowing inference of network rewiring associated with immune phenotypes such as T-cell exhaustion or M2 macrophage polarization (Zhang et al., 2025).

Using such strategies, one study proposed an “immunometabolic” PDAC subtype characterized by coordinated shifts in glycolysis and oxidative phosphorylation, enrichment of immunosuppressive myeloid populations, and reduced sensitivity to conventional iRT—yet with potential vulnerability to combinations targeting metabolic pathways (Bandi et al., 2023; Xue et al., 2026). In PDAC, AI-assisted subtyping may help refine stratification beyond single biomarkers and bulk classifications, and may improve trial enrichment when supported by cohort-level validation.

### 2.2 Transformative insights from digital pathology and spatial multi-omics

AI analysis of whole-slide images has progressed from cell enumeration to quantification of tissue architecture. Convolutional neural networks (CNNs) and vision transformers (ViTs) can characterize cellular composition, morphology, and spatial organization among tumor cells, cancer-associated fibroblasts (CAFs), and diverse immune populations (Cai et al., 2025; Sun et al., 2023). These approaches also enable more objective assessment of tertiary lymphoid structures (TLS)—including presence and maturation—features with prognostic and potentially predictive implications (Le Rochais et al., 2025; Alissa et al., 2025).

Meanwhile, spatial transcriptomics and multiplex imaging platforms (multiplex immunofluorescence, CODEX) generate high-dimensional spatial maps at cellular or subcellular resolution. AI algorithms, especially graph-based models and spatial statistical frameworks, can delineate immune-activated, immune-excluded, and immune-suppressed niches and infer intercellular communication networks (Valanarasu et al., 2026; Lu et al., 2021; Srivastava et al., 2025; Wu et al., 2022). A recurring insight is that cytotoxic T cells may remain functionally constrained near specific stromal architectures that act as physical and biochemical barriers, even when immune infiltration is present. This supports a clinically actionable reframing of RT: not only a cytotoxic modality, but also a potential tool to disrupt immunosuppressive stromal scaffolds and reconfigure immune access.

### 2.3 Dynamic monitoring through radiomics and liquid biopsy

Radiomics extracts high-throughput quantitative features from CT, MRI, and PET–CT and can be integrated by AI to generate noninvasive “virtual biopsy” surrogates of stromal content and immune state (Trebeschi et al., 2019; Huang et al., 2025). Deep learning can also learn latent image patterns directly from raw imaging that correlate with fibrosis severity, tumor purity, and molecular subtype (Tiwari et al., 2025). A prospective study reported that a deep learning–based radiomics model from preoperative CT could predict postoperative immune microenvironment states and recurrence risk in PDAC (Cao et al., 2023). For longitudinal assessment, AI-driven modeling of circulating tumor DNA (ctDNA) can support sensitive detection of molecular remission, early identification of emerging resistant clones, and timely recognition of immune-related adverse events. By integrating serial radiomics and ctDNA trajectories, AI can provide patient-specific response curves that may anticipate outcome earlier and more precisely than RECIST-based evaluation (Montagut and Vidal, 2019).

## 3 AI-driven optimization of immunoradiotherapy synergy: from empirical combinations to intelligent design

Effective iRT is a systems problem: outcome depends on dose, fractionation, timing, sequencing, and spatial targeting, and these variables interact with tumor biology. AI enables an engineering-style workflow—simulation, optimization, and iterative refinement—rather than empirical trial-and-error.

### 3.1 Personalized radiotherapy dose and fractionation design

RT immunomodulation can be “double-edged”: appropriate dosing can promote immune priming, whereas excessive exposure or suboptimal fractionation can activate suppressive pathways and damage effector compartments. Uniform fractionation strategies (standard RT or SBRT) therefore risk mismatching the patient’s TME (Xu et al., 2025). Reinforcement learning (RL) and generative modeling have been proposed to explore individualized schedules, primarily as simulation tools to generate candidate regimens for subsequent clinical testing in PDAC. In a typical RL framework, a virtual TME dynamics model defines the environment; the agent tests candidate dose–time schedules and is rewarded according to modeled immune activation or suppression indices, converging on strategies tailored to a given virtual patient (Cockrell et al., 2022; Van den Ende et al., 2020). Early studies suggest these learned regimens may differ meaningfully from conventional practice. For example, in Treg-enriched settings, models may favor staged, dose-intensified “pulsed” irradiation aimed at preferentially reducing suppressive compartments while preserving effector function (Chen et al., 2024; Chen et al., 2025). Clinically, the most immediate value is to generate biologically plausible schedules for prospective evaluation rather than to replace clinician judgment.

### 3.2 Biologically guided target delineation and dose painting

Traditional target volumes (GTV/CTV) are predominantly anatomy-driven. AI enables integration of functional imaging (e.g., DWI-MRI, dynamic contrast-enhanced MRI, FET-PET) with molecular and pathological information to define biologically informed target volumes (BTVs) (Ertl-Wagner and Wagner, 2023). Models can localize intratumoral subregions associated with hypoxia, high proliferation, or elevated immunosuppressive signaling—features linked to immune resistance (Okano et al., 2021). AI-driven planning can then implement “dose painting,” delivering spatially heterogeneous doses matched to biological subvolumes (Lassau et al., 2020). Importantly, the goal extends beyond maximal cytotoxicity to deliberate immune remodeling (Kedra and Gossec, 2020). Conceptually, ablative doses might be directed toward immune “desert” cores to boost antigen release, while intermediate doses might be applied to immune-infiltrated margins to activate rather than eradicate resident immune cells (Ontaneda et al., 2021). This reframes RT planning as an immunobiology-aware intervention.

### 3.3 Virtual screening of treatment sequencing and combination strategies

The sequencing of ICIs relative to RT—pre-, concurrent, or post-irradiation—can materially affect antigen presentation, priming, expansion, and exhaustion dynamics, yet the optimal schedule is context dependent (Van et al., 2026). Hybrid mechanistic–AI models and systems pharmacology frameworks can construct virtual patient cohorts to simulate large numbers of schedules and combinations (Basile et al., 2019). Such *in silico* experiments can prioritize regimens based on predicted effects across immune activation steps, functioning as “virtual clinical trials” that accelerate hypothesis generation and sharpen real-world trial design (Noor et al., 2023; Dharmasivam et al., 2026), particularly for prioritizing schedules to be tested in PDAC-focused trials.

## 4 Constructing predictive biomarkers and an adaptive therapeutic feedback loop

A practical clinical aim is to use baseline features together with early on-treatment signals to inform reassessment and potential adjustment of iRT, within MDT workflows and with prespecified review timepoints.

### 4.1 Multimodal composite digital biomarkers

Single biomarkers such as PD-L1 expression and tumor mutational burden (TMB) have limited predictive value in PDAC (Noor et al., 2023). AI enables integration of heterogeneous inputs—genomics, digital pathology, radiomics, and clinical laboratory variables—into composite digital biomarkers. A representative model may combine (i) a stromal gene-expression signature, (ii) spatial metrics of CD8^+^ T-cell proximity and interaction with CAFs at the tumor–stroma interface derived from whole-slide images, and (iii) radiomic texture heterogeneity from contrast-enhanced CT. Such multidimensional “digital fingerprints” may better represent the biological determinants of iRT response and improve discrimination between responders and non-responders (Prelaj et al., 2024; Li et al., 2025; Cui et al., 2025).

### 4.2 Dynamic risk prediction and early warning systems

Beyond baseline stratification, AI can incorporate longitudinal changes to enable dynamic risk prediction. By analyzing early on-treatment signals—first post-treatment imaging and temporal shifts in circulating biomarkers—models may forecast longer-term outcomes (PFS/OS) within weeks of initiation (Kale et al., 2024). This supports earlier clinical decisions than conventional radiographic progression criteria (Zeng et al., 2022). For patients predicted to progress early, actionable options include earlier transition to second-line regimens, modification of RT/immunotherapy parameters, or timely referral to trials of novel combinations.

### 4.3 Toward adaptive, intelligent therapeutic systems

A longer-term vision is an integrated, response-guided workflow. At diagnosis, multimodal data generate an individualized initial iRT strategy. During treatment, serial imaging, ctDNA, patient-reported outcomes (PROs), and toxicity profiles are collected at prespecified intervals and fed back into the model to update risk estimates over time. The system then outputs clinically interpretable recommendations—e.g., selective SBRT escalation based on evolving radiomic signatures, or addition of targeted agents when ctDNA reveals emerging resistant clones (Tyler et al., 2020; Jiang et al., 2023). This approach emphasizes structured reassessment and clinician oversight when using model outputs to support treatment planning.

## 5 Discussion: controversies, challenges, and a future roadmap

AI-driven iRT offers substantial promise, but translation is constrained by conceptual divergence, technical barriers, and evaluation gaps.

### 5.1 Academic controversies and divergent conceptual pathways

A key debate concerns what AI should optimize. The “immune sensitivity optimization” view argues that modest iRT efficacy in PDAC largely reflects inadequate patient selection and suboptimal regimen design rather than biological impossibility (Li et al., 2022; Volkmar et al., 2025). Here, AI may support refinement of stratification and exploration of dose/fractionation and sequencing parameters within current tools (RT and approved ICIs), in line with available clinical and translational data.

In contrast, the “paradigm innovation” view holds that PDAC immunosuppression is deeply entrenched in its TME, making the ICI–RT backbone intrinsically insufficient (Principe et al., 2021; Avanzo et al., 2021). In this framework, AI may help integrate multimodal data to support rational selection of combination partners beyond ICI–RT, including stromal- and myeloid-targeted approaches, cellular therapies, and vaccines (Huang et al., 2022; Nimgaonkar et al., 2023). AI thus functions not only as an optimizer but as a designer of biologically coherent combination strategies.

### 5.2 Core challenges limiting clinical translation

Despite rapid conceptual and technical progress, several practical constraints continue to slow the clinical uptake of AI-guided iRT in PDAC. A recurrent bottleneck is the scarcity of large, high-quality, deeply annotated multimodal datasets. In real-world settings, relevant data are often fragmented across institutions and locked within “data silos,” while the most informative modalities—spatial multi-omics and high-resolution digital pathology—remain expensive to generate and labor-intensive to annotate. Heterogeneity in acquisition protocols, staining platforms, and sequencing pipelines further introduces batch effects that can erode model robustness and limit cross-center generalizability.

Equally important is the issue of clinical trust. Many state-of-the-art deep learning systems operate as opaque models, and recommendations without biologically plausible explanations are difficult to justify in high-stakes decision-making. This interpretability gap is not only a barrier to adoption; it also restricts the ability of AI models to generate mechanistic insights that could guide rational trial design or identify actionable resistance pathways.

Finally, the current evidence base is still dominated by retrospective analyses or internal validation cohorts, where overfitting and optimistic performance estimates remain a persistent concern. Embedding AI into the operational complexity of iRT workflows—and demonstrating that AI-guided decisions translate into survival gains through prospective trials—has been comparatively rare. Where feasible, evaluation should distinguish internal, temporal, and external validation, and reporting should include calibration and clinical utility in addition to discrimination. Deployment also requires attention to dataset shift/model drift with prespecified monitoring strategies. Traditional randomized controlled trial designs also struggle to evaluate interventions that are adaptive, individualized, and continuously updated over time, creating a mismatch between how AI systems function and how clinical evidence is typically generated.

### 5.3 Future directions and strategic roadmap

Closing the translational gap will require coordinated progress across data infrastructure, modeling strategy, and trial methodology. On the data side, multicenter prospective efforts are needed to collect standardized biospecimens, imaging, pathology, and clinical variables across the full iRT trajectory, with harmonized protocols that reduce inter-site variability. Privacy-preserving approaches—particularly federated learning—can help enable cross-institution model development without sharing raw data, offering a practical route around regulatory barriers while directly addressing fragmentation.

On the modeling side, interpretability must be treated as a design requirement rather than an afterthought. Explainable AI approaches such as attention-based attribution, SHAP, and LIME can provide transparency, but their outputs need to be aligned with clinically meaningful concepts and validated against biological ground truth. Hybrid frameworks may be especially valuable here: physics-informed neural networks (PINNs) and related strategies can embed radiobiological and pharmacokinetic constraints into learning systems, improving robustness, extrapolation, and clinician confidence—particularly when data are limited or shift across settings.

Evaluation strategy also needs to evolve. Adaptive platform trials, umbrella designs, and basket frameworks are better suited than conventional RCTs to test AI-enabled, response-guided interventions. In such trials, AI can function as a stratification and decision-support engine, allowing patients to be assigned—and when appropriate, reassigned—based on baseline predictions and early on-treatment signals. This design more closely matches the dynamic nature of AI-guided therapy and can accelerate the generation of clinically credible evidence while remaining consistent with the principles of precision oncology.

## 6 Conclusion

AI is poised to catalyze a shift in advanced PDAC management. By integrating multimodal data, AI can move iRT from population-averaged, empirically combined regimens toward precisely engineered and dynamically adaptive strategies aligned with each tumor’s biological architecture. This enables more faithful visualization of TME heterogeneity, earlier anticipation of response, and rational personalization of synergistic regimens beyond the reach of conventional design.

Despite unresolved challenges—data availability and quality, interpretability, prospective validation, and ethical governance—these barriers define an actionable roadmap. Continued progress will depend on sustained interdisciplinary collaboration, construction of robust and equitable data ecosystems, innovation in adaptive trial methodology, and explicit commitment to transparency and fairness in AI development. Ultimately, AI-enabled iRT has the potential to convert the long-envisioned promise of precision medicine into tangible survival benefits for patients with advanced PDAC, while signaling a broader transition toward data-driven, dynamically informed cancer therapy.
